# Glucocorticoid Receptor Signaling in Diabetes

**DOI:** 10.3390/ijms222011173

**Published:** 2021-10-16

**Authors:** Ioanna Kokkinopoulou, Andriana Diakoumi, Paraskevi Moutsatsou

**Affiliations:** Department of Clinical Biochemistry, Medical School, National and Kapodistrian University of Athens, University General Hospital “ATTIKON”, 12462 Athens, Greece; iwanna-k@med.uoa.gr (I.K.); adiakoum@biol.uoa.gr (A.D.)

**Keywords:** glucocorticoids, glucocorticoid receptor, GR, diabetes mellitus, stress

## Abstract

Stress and depression increase the risk of Type 2 Diabetes (T2D) development. Evidence demonstrates that the Glucocorticoid (GC) negative feedback is impaired (GC resistance) in T2D patients resulting in Hypothalamic-Pituitary-Adrenal (HPA) axis hyperactivity and hypercortisolism. High GCs, in turn, activate multiple aspects of glucose homeostasis in peripheral tissues leading to hyperglycemia. Elucidation of the underlying molecular mechanisms revealed that Glucocorticoid Receptor (GR) mediates the GC-induced dysregulation of glucose production, uptake and insulin signaling in GC-sensitive peripheral tissues, such as liver, skeletal muscle, adipose tissue, and pancreas. In contrast to increased GR peripheral sensitivity, an impaired GR signaling in Peripheral Blood Mononuclear Cells (PBMCs) of T2D patients, associated with hyperglycemia, hyperlipidemia, and increased inflammation, has been shown. Given that GR changes in immune cells parallel those in brain, the above data implicate that a reduced brain GR function may be the biological link among stress, HPA hyperactivity, hypercortisolism and hyperglycemia. GR polymorphisms have also been associated with metabolic disturbances in T2D while dysregulation of micro-RNAs—known to target GR mRNA—has been described. Collectively, GR has a crucial role in T2D, acting in a cell-type and context-specific manner, leading to either GC sensitivity or GC resistance. Selective modulation of GR signaling in T2D therapy warrants further investigation.

## 1. Introduction

### 1.1. Stress, Depression and Type 2 Diabetes

Diabetes Mellitus (DM) is a group of chronic and heterogeneous metabolic disorders characterized by high blood glucose levels (hyperglycemia) that result from absolute or relative insulin deficiency [1]. DM is a common and fast-growing disease with high morbidity and mortality, mainly due to macro-(atherosclerosis) and micro-vascular (diabetic nephropathy, retinopathy, and neuropathy) complications [2]. Although DM can be classified into two main categories; early-onset autoimmune form (Type 1 Diabetes; T1D) and late-onset non-autoimmune form (Type 2 Diabetes; T2D), there are also additional subtypes, including monogenic (maturity-onset diabetes of the young and neonatal diabetes) and gestational diabetes [1]. T2D accounts for approximately 90–95% of all diagnosed cases of DM and it is considered a complex and multifactorial disease caused by a combination of genetic and environmental risk factors [3]. Genome Wide Association Studies (GWAS) have revealed numerous genetic loci contributing to the risk of developing the disease while environmental factors, including physical inactivity, and an unhealthy diet, lead to increasing weight gain and therefore to the risk of development T2D [4,5]. Increasingly, epidemiological studies have revealed that depression as well as psychological stress —particularly chronic work stress and early life adversity—are risk factors for T2D development [6,7,8]. Depression, a stress-related disorder, has been shown to increase the risk for progressive insulin resistance and incident of T2D [9,10,11,12,13]. Comorbid depression in T2D patients has been associated with poor glycemic control and cardiovascular complications [14]. In addition, a positive association between chronic physiological stress and incident of T2D has been revealed from animal and epidemiological studies, and human laboratory stress trials [15,16,17,18,19]. It has been hypothesized that an overactive Hypothalamic-Pituitary-Adrenal (HPA) axis may be the biological link between depression and T2D-associated metabolic abnormalities [20]. This hypothesis has been elucidated in many studies as demonstrated in the following paragraph.

### 1.2. Hypothalamic-Pituitary-Adrenal Axis Hyperactivation and Hypercortisolism in Type 2 Diabetes Mellitus

The HPA axis is the central stress response system. The stress signal causes the hypothalamus to secrete Corticotropin Releasing Hormone (CRH) which in turn induces the anterior pituitary gland to produce Adrenocorticotropic Hormone (ACTH). ACTH stimulates the adrenal glands to produce increased levels of Glucocorticoids (GCs). High cortisol levels, the predominant endogenous GC, act negatively on CRH and ACTH secretion, terminating the stress response through a negative feedback loop [21]. GCs regulate a variety of physiologic processes in central nervous system (crucial in stress response) and peripheral tissues while their availability is regulated at a tissue or cellular level [22,23]. The majority of circulating GCs are bound to Corticosteroid Binding Globulin (CBG) and albumin in order to be maintained in an inactive form. Only a small percentage of systemic GCs are free and bioactive [24]. At the cellular level, GC availability is sustained by the tissue-specific metabolic enzymes 11β-hydroxysteroid dehydrogenases (11-β-HSDs), which catalyze the interconversion of active GCs. More specifically, 11β-HSD2 rapidly inactivates GCs, converting cortisol to cortisone, while 11β-HSD1 facilitates the conversion of inactive cortisol to bioactive cortisol [25]. Circulating GC levels fluctuate naturally in an ultradian and circadian fashion, reaching their zenith in the early morning and their nadir in the late evening. The circadian secretion of cortisol is generated by the evolutionary conserved circadian clock system, which consists of central and peripheral components [26,27].

GCs, via their cognate receptor, the Glucocorticoid Receptor (GR), mediate their negative feedback mechanism. GR is the key mediator of HPA axis negative feedback [28]. HPA hyperactivation, hypercortisolism, and disturbances in diurnal cortisol release have been considered the biological link between stress, depression, and T2D [20]. Activated HPA axis and chronic hypercortisolism is known to lead to metabolic and immune disturbances such as hyperglycemia, hyperlipidemia, insulin resistance and inflammation, states known to characterize T2D patients [29,30]. The relationship among neuroendocrine dysfunction, HPA hyperactivity and T2D has been evaluated in patients with T2D, measuring cortisol levels in plasma and 24-hour urine as well as by using the Dexamethasone Suppression Test (DST). In particular, patients with T2D exhibit non-suppression of HPA axis activity following GC administration, suggesting that GC negative feedback is impaired in these patients [31,32,33,34]. Elevated ACTH levels [35] and increased urinary free cortisol levels [36] have also been described in diabetic patients. Champaneri et al. also revealed elevated cortisol throughout the day in women with diabetes [37]. Diabetic complications, such as diabetic neuropathy, have been associated with a specific and persistent increase in the activity of the HPA axis [35]. The degree of severity of several clinical measures of T2D, including fasting blood, urinary and postprandial glucose, glycosylated hemoglobin, and systolic and diastolic blood pressures has been also correlated with cortisol concentrations [38]. Furthermore, T2D subjects with diabetic complications exhibit enhanced HPA activity and the degree of cortisol secretion has been related to the presence and number of diabetes complications [31].

Despite that DST is widely used for the evaluation of HPA function, this assay is difficult to implement in large population studies [39]. On the other hand, salivary cortisol collection consists of a noninvasive method for the measuring of free cortisol and allows for a valid assessment of the HPA axis [20]. Salivary cortisol collection is used to construct the diurnal salivary cortisol curve which includes several cortisol parameters, including wake-up cortisol levels, bedtime cortisol levels, cortisol levels 30–45 min after awakening (Cortisol Awakening Response; CAR), total area-under-the-curve (AUC_total_), and the total area-under-the-curve in respect to increase (AUC_i_) [40]. In particular, CAR reflects the organism’s response to the natural stressor of awakening, and it is considered an index of HPA axis basal activity and stress reactivity [41,42]. AUC_i_ reflects total cortisol levels during the awakening response while AUC_total_ reflects total cortisol level during the day [20,43].

Evidence has shown that T2D patients exhibit multiple disturbances in salivary cortisol curve parameters (measures of HPA axis function) supporting further HPA axis dysfunction in T2D patients. Data derived from saliva cortisol concentrations reveal that T2D patients exhibit flattened circadian cortisol profile [44], flatter slope across the day, higher bedtime cortisol values [17] and a blunting of the CAR [45]. Interestingly, the association between salivary cortisol curve parameters and T2D seems to be sex-specific; diabetic men showed greater CAR compared to non-diabetic men, while diabetic women had significant elevated night-time cortisol levels compared to non-diabetic women [46]. On the other hand, Spanakis et al. found no association between T2D and changes in daily salivary cortisol curve features in a longitudinal 6 year cohort study and therefore they supported no HPA dysregulation in T2D [47]. Bellastella et al. assessed the diurnal salivary and plasma cortisol variations in T2D patients for evaluating whether glycemic control and glycemic oscillations may affect cortisol concentrations [48]. Their study revealed a significant correlation between late night serum cortisol and indices of intra-day glycemic variability only in the group of T2D patients. On the other hand, late night salivary cortisol did not correlate with any indices of glucose variability, revealing that late night salivary rather than plasma cortisol may give information on the dynamics of adrenal function in T2D [48]. Despite the conflicting results, which may be attributed to the study samples or the collection time and number of cortisol samples, diabetes seems to be associated with a flatter diurnal cortisol curve, blunted CAR, and flatter slope across the day, revealing HPA hyperactivity in T2D patients. Given that HPA hyperactivity and chronic hypercortisolism lead to metabolic and immune disturbances in peripheral tissues, such as hyperglycemia, hyperlipidemia, insulin resistance, and inflammation [29,30], the aforementioned data support that HPA hyperactivity and excess cortisol release may be the biological link between stress, depression, and diabetes [20].

Since cortisol mediates its effects via GR, in the following paragraphs we summarize the current knowledge which uncovers the key role of GR in regulating crucial steps of glucose homeostasis and insulin signaling in metabolic tissues. Although the role of GR in hyperglycemia and other metabolic abnormalities is well-characterized in peripheral metabolic tissues such as liver, skeletal muscle, adipose tissue, and pancreas, data about GR function in brain and immune cells in T2D are limited. The main aim of this review is to present and discuss in detail recent evidence regarding GR signaling in Peripheral Blood Mononuclear Cells (PBMCs) in T2D and its implication in GR function and HPA axis negative feedback in this disease. In addition, GR polymorphisms which have been associated with metabolic disturbances in T2D and micro-RNAs-known to target GR mRNA are also described. To help understand the mechanistic discussions presented in this review, a brief description of the GR subtypes and their modes of action are included below.

## 2. Glucocorticoid Receptor

### 2.1. Mechanism of Action

The action of GCs is mediated mainly by an intracellular protein, the GR. GR is a DNA-binding transcription factor belonging to the nuclear receptor superfamily, which is ubiquitously expressed. GR is encoded by *NR3C1* gene, which is located on chromosome 5 (5q31). Its structure is characterized by the presence of three functional domains: the N-terminal transactivation domain (NTD), the central DNA-binding domain (DBD), and the C-terminal ligand-binding domain (LBD) [49,50,51]. Full-length GRα is the classic mediator of the GC effects, but four additional splice variants have been identified; GRβ, GRγ, GR-A, and GR-P [52]. GRβ acts as a natural negative inhibitor of the GRα isoform [53]. In its unliganded state, GRα, the predominant GR isoform, resides in the cytoplasm and associates with chaperone proteins (heat shock proteins 70/90, p23 and immunophilin FKBP51). Upon GC binding, GRα translocates to the nucleus where it homodimerizes, and binds to Glucocorticoid Response Elements (GREs) in the promoter region of target genes, mediating their regulation of expression (negatively or positively) [22]. The GC/GRα complex also interacts with proinflammatory transcription factors, including Nuclear Factor κB (NF-κB), Activator Protein-1 (AP-1), and Signal Transducer and Activator of Transcription 5 (STAT-5), regulating the expression of their target genes [54,55,56]. GCs via the GR-GRE transactivation mechanism exert detrimental effects on metabolism while via the GR-NFκB trans-repression mechanism mediate their beneficial anti-inflammatory effects [57]. However, GR-GREs dependent transactivation is also essential in the anti-inflammatory activities of GR [57].

GR function can be modified by post-transcriptional (microRNAs) and post-translational modifications, including methylation, acetylation, nitrosylation, sumoylation, ubiquitination, and phosphorylation, affecting protein’s stability, localization, interaction with other proteins, and ligand response [58,59,60,61,62,63,64]. Studies have shown that the circadian rhythm-related transcription factor CLOCK physically interacts with the LBD of the GR and suppresses the GR-induced transcriptional activity by acetylating multiple lysine residues in the receptor [60]. This post-translational modification attenuates the binding of GR to GREs, leading to a cyclic GR-mediated transcriptional regulation. 

The cellular response to GCs exhibits variability both in magnitude and in specificity of action, resulting in considerable heterogeneity in GC sensitivity. Multiple mechanisms, including pre-receptor ligand metabolism, receptor isoform expression, tissue- or cell- specific factors, and molecular heterogeneity of GR proteins are responsible for diversity and specificity in response to GCs [23]. Alterations in the molecular mechanisms of GR action may impair or enhance GC signal transduction and thus alter tissue sensitivity to GCs [65]. In particular, GR phosphorylation and acetylation may affect GR responsiveness to GCs. Acetylated GR exhibits lower affinity for NF-κB, and therefore cannot repress NF-κΒ induced inflammatory gene transcription. Histone Deacetylase 2 (HDAC2) deacetylates GR, enabling the association of GR with NF-κB [66]. Specific knockdown of HDAC2 by RNA interference led to GC insensitivity and inhibited the association between GR and NF-κB while overexpression of HDAC2 in GC-insensitive alveolar macrophages from patients with chronic obstructive pulmonary disease is able to restore GC sensitivity [66]. Phosphorylation of GR by p38 mitogen-activated protein kinase alters GR function, also resulting in GC resistance [67,68]. High GRβ expression has been associated with GC resistance in GC resistant cases of numerous diseases while GRα/GRβ expression ratios also provide an indication of GC sensitivity [69,70].

Increasing evidence supports that Reactive Oxygen Species (ROS) alter GR structure and impair its function. Tienrungroj et al. were the first to suggest that treatment of the transformed GR with hydrogen peroxide (H_2_O_2_) promotes the formation of disulfide bonds and inhibits the ability of the receptor to bind to DNA [71]. Later, Silva et al. provide direct evidence that intra- and intermolecular disulfide bonds can form within GR after in vitro oxidation with tetrathionate and inhibit GR activation [72]. This oxidative inhibition of the GR was reversed by the thiol-reducing agent dithiothreitol [72]. Hutchison et al. revealed that the effect of H_2_O_2_ is on sulfhydryl groups within the zinc fingers of the GR DBD [73] and particularly, H_2_O_2_ induce the formation of a disulfide bond between two close cysteine residues [74]. More evidence for the redox modulation of GR function in cell cultures and in vivo has been obtained [75,76]. Makino et al. have extensively investigated the role of redox modulation in GC regulation. Initially, they provide evidence showing that cellular GC responsiveness is coordinately modulated by redox state and Thioredoxin (TRX) levels [77]. The same group revealed in CHO cells expressing the human GR that H_2_O_2_ treatment decreased the ligand-binding and transcriptional activity of the receptor [78]. More importantly, they showed that an association between TRX and GR DBD occurs in the nucleus to restore the transactivational GR function under conditions of oxidative stress [79]. In addition, the same group showed that oxidative conditions impaired both ligand dependent and independent nuclear translocation of the GR [61]. Collectively, increased ROS and oxidative stress impair GR function and promote a GR resistant state.

Rapid nongenomic GC actions have been also defined, mediated by activation of signal transduction pathways, such as Phosphoinositide 3-Kinase (PI3K), Protein Kinase B (AKT), and Mitogen-Activated Protein Kinases (MAPKs) [80,81,82,83,84]. Membrane-bound GR (mGR) has also been identified and it has been related to intracellular signaling pathways mediated by G-protein-coupled receptors [85]. Moreover, the identification of GREs in mitochondrial genome and the detection of GR in mitochondria of different cell types enhance the additional role of GR in mitochondrial gene expression and energy metabolism [86,87].

There are different approaches in testing GR function in vitro and in vivo. Among them are in vitro assessments of the GR function by analyzing immune cells after GC treatment as well as in vivo assessments observing diurnal levels of cortisol, CAR, or DST [88]. DST evaluates the GC-mediated negative feedback on the HPA axis (GC-sensitivity in central nervous system) while the GC-sensitivity in immune system is assessed by appraisal of the trans-repressive effects of Dexamethasone (Dex) in Lipopolysaccharide (LPS)-induced cytokine levels or by evaluating the trans-activating effects of Dex on GR target gene (*GILZ* and *FKBP5*) expression in PBMCs. The higher the concentration of Dex required to induce these mRNA levels or to reduce cytokine levels the lower is the GC-sensitivity [88].

### 2.2. Glucocorticoid Receptor and Metabolism 

Τhe detrimental effects of GCs on metabolism in peripheral tissues is well established and has been under extensive study for several decades. Patients exposed to pharmacological doses of GCs for therapeutic purposes demonstrate metabolic dysregulations in carbohydrate, lipid, and protein metabolism [89]. Similarly, patients suffering from Cushing’s syndrome exhibit impaired glucose intolerance, suggesting that some individuals are prone to develop diabetes in response to high GC levels [90,91]. GC excess, either endogenous or exogenous, results in central obesity, muscle atrophy, fatty liver, hypertension, hyperglycemia, dyslipidemia, and insulin resistance [92,93,94,95]. Mounting evidence has shown the mechanisms underlying the effect of GCs on metabolism. Briefly, GCs regulate key enzymes of glucose metabolism in the liver, skeletal muscle, adipose tissue, and pancreas. In liver, cortisol induces lipogenesis and gluconeogenesis, as well as the release of glucose by hepatocytes [96,97]. In the muscle, GCs inhibit glucose uptake and glycogen synthesis, and enhance proteolysis [95,98,99]. In adipose tissue, GCs promote pre-adipocyte differentiation into adipocytes, and hypertrophy in abdominal fat, while increase lipolysis and reduce Lipoprotein Lipase (LPL) activity in peripheral fat [100,101]. In pancreas, GCs regulate the secretion of glucagon and insulin, two hormones that play a pivotal role in the regulation of blood glucose levels [102,103]. GCs have been shown to inhibit glucose-stimulated insulin release, impair β-cell glucose uptake, and reduce calcium fluxes [104]. Clear evidence has demonstrated that GCs inhibit insulin secretion and induce insulin resistance in peripheral tissues [105,106].

At the molecular level, a great number of studies have revealed the critical role of the hepatic GC–GR axis for glucose homeostasis. The key gluconeogenic enzymes involved are tightly controlled by GCs. GCs promote gluconeogenesis mainly through activation of the transcription of genes encoding enzymes in the gluconeogenic pathway. Various genes of gluconeogenic pathway seem to be direct regulatory targets of GR action and are characterized by the presence of GREs, including *Tyrosine Aminotransferase (TAT), Phosphoenolpyruvate Carboxykinase (PEPCK), Glucose-6-Phosphatase (G6PC), G6P Transporter (SLC37A4)* and *6-Phosphofructo-2-Kinase/Fructose-2,6-Biphosphatase 1 (PFKFB1)* [107,108,109,110,111]. In addition, GR regulates the expression and activity of many genes involved in these metabolic processes via heterodimerization with other transcription factors including AP-1, NF-κB, and STAT-5 [54,55,56]. Regarding the molecular mechanisms involved in glucose metabolism, GCs induce expression but inhibit the translocation to the membrane of the Glucose Transporter Type 4 (GLUT4) in both muscles and adipose tissue via a GR-mediated mechanism [112]. GCs also inhibit glucose oxidation by inducing the expression of *Pyruvate Dehydrogenase Kinase 4 (PDK4)*, a GR primary target gene [113]. In addition, a great number of potential GR primary target genes that can supress insulin action have been identified in mouse myotubes, including *Phosphoinositide-3-Kinase Regulatory Subunit 1 (PIK3R1)* [114].

Several studies in diabetic animal models have revealed the role of GR in hyperglycemia and diabetes development. Importantly, blocking the GR with selective GR antagonists specific to liver or adipose tissue leads to a decrease in glucose levels in diabetic rodents [115,116,117,118], supporting a role of GR in hyperglycemia. Increased expression of GR has been observed in the liver of diabetic mice, while treatment with the GR antagonist-RU486-reversed the increase in the expression and attenuated the phenotype of T2D, supporting that the activation of GR may contribute to the development of T2D [119]. Mice with inactivation of the GR gene exhibit hypoglycemia after prolonged starvation due to reduced expression of genes involved in gluconeogenesis [120]. The absence of GR in hepatocytes limits the development of hyperglycemia in streptozotocin-induced diabetes mellitus due to down-regulation of gluconeogenic enzyme genes, suggesting that GR is involved in the development of diabetic hyperglycemia [120]. Xu et al. determined that 5-chloro-N-(4-chloro-3-(trifluoromethyl)phenyl)thiophene-2-sulfonamide (FX5), a non-steroidal GR inhibitor, effectively reduced gluconeogenesis and improved glucose homeostasis in diabetic model mice, either directly by antagonizing GR or via the GR/HNF4α/miR122–5p signaling pathways [121]. Chen et al. investigated the expression levels of gluconeogenetic enzymes, *PCK1* and *G6PC*, as well as *GR* mRNA levels in diabetic rats [122]. Their study revealed higher expression levels of *PCK1, G6PC* and *GR* mRNA in the liver of diabetic rats, indicating that diabetes increases the GC activity and gluconeogenesis. After gossypol treatment, an 11β-HSD1 inhibitor, the expression levels of *PCK1, G6PC* and *GR* mRNA were significantly reduced, revealing that gossypol could be an effective treatment for T2D [122].

Interestingly, in a recent report, Aylward et al. cultured primary pancreatic islet in normal conditions after Dex treatment (high and low dose) and they profiled gene expression and islet accessible chromatin, using RNA-sequencing (RNA-seq) and Assay for Transposase-Accessible Chromatin using sequencing (ATAC-seq), respectively [123]. Their study revealed upregulated chromatin sites which were enriched for GR binding sites as well as upregulated genes mainly related with ion transport and lipid metabolism. Conversely, downregulated chromatin sites and genes were enriched for inflammatory, stress response, and proliferative processes. Furthermore, the same study revealed that genetic variants associated with glucose levels and T2D risk were enriched in glucocorticoid-responsive chromatin sites, while a likely causal variant at the 2p21 locus had glucocorticoid-dependent allelic effects on beta cell enhancer activity and affected the expression of the transcription factors *SIX2* and *SIX3* [123]. In summary, the above in vivo and in vitro studies demonstrate that GR is the key transcription factor which regulates genes involved in carbohydrate and lipid metabolism and mediates the detrimental effects of GCs on metabolism, pointing out its role in diabetes development. 

## 3. Glucocorticoid Receptor Signaling in Immune Cells in Type 2 Diabetes Mellitus

Despite the increasing evidence focused on a better understanding of GR signaling in diabetes in peripheral tissues, there are limited data regarding measurement of key steps in GR signaling in brain tissue and its involvement in HPA axis hyperactivity in T2D. Due to limited access in brain GRs and because GRs are similarly regulated in the brain and immune cells [124,125], Panagiotou et al. assessed GC sensitivity as well as key GR signaling parameters (*GRβ*, pGR-S211, *GILZ1*, and *FKBP5*) in PBMCs of T2D patients [126]. Their possible association with measures of glycemia, lipidemia, inflammation, and energy metabolism was also assessed. In particular, Panagiotou et al. measured two important regulators of GR function such as the bioactive phosphor(p)GRs-S211 and the abundance of *GRβ* isoform. For the evaluation of GC-sensitivity in PBMCs of T2D patients, Panagiotou et al. assessed the expression levels of the GR-transactivated genes *GILZ* and *FKBP5*, in basal condition and after Dex treatment. The levels of Interleukin 1β (IL-1β), which are regulated via a GR-trans-repressed mechanism, were also assessed. Their study revealed that T2D patients exhibit a significant decrease in the nuclear bioactive pGR-S211 protein levels, suggesting impaired GR signaling in PBMCs of T2D patients [126]. PBMCs of T2D patients also exhibited higher *GRβ* mRNA expression levels and lower *GRα/β* ratio, pointing out reduced GC sensitivity. Moreover, the decreased expression levels of *GILZ* and *FKBP5* and increased IL-1β protein levels at the basal state in T2D patients compared to healthy individuals, further supported the deficiency of GR signaling and revealed GC-resistance in PBMCs of T2D patients. In addition, T2D patients exhibited lower responses to Dex-induced effects on *GILZ* and *FKBP5* expression compared to healthy individuals, further supporting the presence of GC resistance in PBMCs of T2D patients [126]. The negative association between *GRβ* and basal *GILZ* mRNA expression implicates that the reduced GC sensitivity may possibly be due to increased *GRβ* expression levels. For the evaluation of HPA axis activity, salivary cortisol parameters were also determined, revealing blunted CAR, flattened salivary diurnal curve, and increased total diurnal cortisol secretion in T2D patients. Importantly, the impaired GR signaling and the reduced saliva cortisol profile in T2D patients were associated with hyperglycemia, hyperlipidemia, increased inflammation, low adiponectin, and impaired energy metabolism [126]. Collectively, Panagiotou et al., measuring numerous parameters of GR signaling, revealed an impaired GR function and GC resistance in PBMCs of T2D patients as well as HPA axis dysfunction further associated with diabetes-associated abnormalities. Given that GR changes in PBMCs parallel those in the brain, Panagiotou et al. findings implicate that the defective GR signaling and GC resistance in PBMCs may reflect a GR dysfunction and GC-resistance in central nervous system in T2D. To this direction, GR may be the biological link between the HPA axis hyperactivity and the diabetes-associated metabolic abnormalities and inflammation in T2D. Interestingly, impaired GR signaling in PBMCs, low CAR, hypercortisolism, unfavorable metabolism and increased inflammation are also characteristics of patients with major depression, a disease known to be characterized by HPA hyperactivity and GR defects in the brain [126].

Carvalho et al. also analyzed the GC sensitivity using LPS-induced IL-6 production in whole blood cells of people with diabetes and healthy controls. Their study revealed that whole blood cells from diabetes participants were less capable of producing IL-6 after LPS stimulation, when compared with healthy controls [127]. Interestingly, Carvalho et al. supported increased GC-peripheral sensitivity in T2D patients, due to lower Dex concentration needed to inhibit LPS-induced IL-6 in whole blood cells of T2D patients, as compared to controls [127]. The conflicting data between Carvalho et al. and Panagiotou et al. studies regarding the evaluation of GC sensitivity in T2D could be attributed to different GR sensitivity assay protocols as well as in different measures used for the evaluation of GC-sensitivity. For the assessment of GC-sensitivity Carvalho et al. used mitogen stimulated blood cells and measured the Dex-induced inhibition of cytokine production (ex vivo). Panagiotou et al. evaluated the GC-sensitivity in non-stimulated cells since the artificial immune-stimulation (mitogen-induced) might cause alterations in the GR number or the GR expression [128]. Moreover, there are mechanistic differences between the ex vivo tests which measure GC-sensitivity based on either GR-gene stimulation (such as *GILZ* and *FKBP5*) or GR-gene repression (such as IL-6) [129]. The aforementioned alterations of GR signaling parameters and the evaluation of GC response in immune cells of T2D patients are summarized in Table 1.

Joseph et al. had previously hypothesized that an impaired brain GR function (GR is the key mediator of HPA axis negative feedback) could be the critical link between hypercortisolism and T2D [20]. In support to their hypothesis, Panagiotou et al. [126] provide evidence about an impaired GR function in immune cells in T2D and implicate that similarly to immune cells a reduced GR function may also be present in brain. Campbell et al. have revealed a gradual increase in basal HPA axis activity in sedentary male diabetic rats probably due to reduced negative feedback regulation of the HPA axis as a result of diminished hippocampal GR content (GC resistance), supporting further a defective GR signaling in brain [130]. 

**Figure 1:**
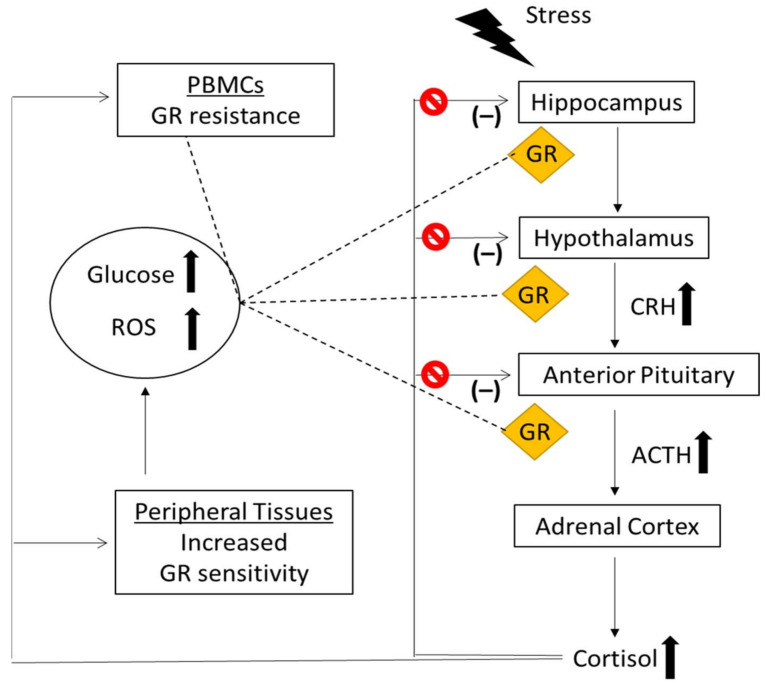
A proposed model for the relationship between hypercortisolism and hyperglycemia in T2D. Cur-rent evidence supports that T2D patients exhibit an impaired negative feedback of the HPA axis (GC resistance) resulting in HPA axis hyperactivity and hypercortisolism. Excess of cortisol release exert detrimental effects on glucose metabolism and insulin signaling-via GR-in GC sensitive pe-ripheral tissues (liver, skeletal muscle, adipose tissue, and pancreas) resulting in hyperglycemia. In contrast to increased GR sensitivity in metabolic tissues, impaired GR signaling and GR resistance in PBMCs of T2D patients further associated with hyperglycemia, hyperlipidemia, and inflammation have been identified. Given that GR is similarly regulated in brain and immune cells, the above data give rise to hypothesis that an impaired brain GR function may be the biological link among stress, HPA axis hyperactivity, hypercortisolism and hyperglycemia. Specifically, in T2D patients, the hyperglycemia-induced production of ROS and oxidative stress may impair GR function and promote a GR resistant state in HPA axis negative feedback and hypercortisolism which in turn may lead to detrimental effects on peripheral metabolic tissues (hyperglycemia), creating a vicious cycle. 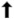 increased levels, 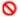 impairment (blockade) of the HPA axis negative feedback, GR; Glucocor-ticoid Receptor, CRH; Corticotropin Releasing Hormone, ACTH; Adrenocorticotropic Hormone, PBMCs; Peripheral Blood Mononuclear Cells, ROS; Reactive Oxygen Species.

It is important to mention that hyperglycemia resulting from streptozotocin-induced diabetes increases the activity of the HPA axis, while normalizing glucose with either insulin or phloridzin corrects this hyperactivity [131,132]. Moreover, ingestion of glucose increases serum and salivary cortisol levels in healthy adults [133,134,135,136], suggesting that glucose can promote a GC resistance state [136]. The signaling pathways that are directly triggered by hyperglycemia, probably through alterations in CLOCK genes and acetylation of GR or due to the hyperglycemia-induced production of ROS and oxidative stress may result in a GR resistant state [136,137,138]. Based on above and bearing in mind that ROS, oxidation status and alterations in CLOCK genes impair GR function, one may propose the following model for the relationship between stress, hypercortisolism, hyperglycemia and GR resistance in immune and brain cells. According to this, an impaired brain GR signaling results in HPA hyperactivity, hypercortisolism, and hyperglycemia. Hyperglycemia-induced production of ROS and oxidative stress, in turn, impair GR function in immune cells as well as in brain cells resulting in further blunting of HPA axis and hypercortisolism in T2D, creating a vicious cycle (Figure 1).

## 4. Glucocorticoid Receptor Polymorphisms in Type 2 Diabetes Mellitus

Several mutations and polymorphisms in the gene coding for the GR have been described and they have been associated with altered GC sensitivity and changes in body composition and metabolic parameters [23]. The most well-studied polymorphisms of *NR3C1* gene are ER22/23EK, BclI, N363S, Tth111, GR-9β, and 22 C/A. ER22/23EK (rs6189 and rs6190) corresponds to two linked polymorphisms in codons 22 and 23 of exon 2. These Single Nucleotide Polymorphisms (SNPs) are fully linked and are associated with GC resistance while carriers of the ER22/23EK polymorphism exhibit lower total cholesterol levels, and lower fasting insulin levels [139]. Furthermore, only in women, the ER22/23EK polymorphism has been associated with reduced first-phase glucose-stimulated insulin secretion and disposition index, which are considered parameters of β-cell function [140]. Male carriers of ER22/23K polymorphism were also found to have beneficial body composition at young-adult age, as well as greater muscle strength [141].

Tth111I (rs10052957) is a restriction fragment length polymorphism located in the 5′ flanking region of the *NR3C1*. Carriers of the Tth111I polymorphism have been associated with elevated basal and bedtime levels of salivary cortisol [142]. Evidence of partial linkage of Tht111I to the ER22/23K polymorphism is associated with decreased GC sensitivity, lower fasting insulin levels, and lower total and Low-Density Lipoprotein (LDL) cholesterol levels [143].

BclI polymorphism (rs41423247) is a C to G substitution change located in the intron 2, 646 nucleotides downstream from exon 2. Homozygous carriers of the BclI polymorphism have been associated with increased body weight, Body Mass Index (BMI), abdominal obesity, fasting plasma glucose, insulin, index of insulin resistance (HOMA), and deficient GR function [144,145,146]. Geelen et al. have also revealed higher total body fatness, higher BMI, and increased insulin resistance in homozygotes of BclI polymorphism [147]. BclI polymorphism has also been associated with increased sensitivity to GCs [148]. Patients with Addison’s disease carrying the polymorphic BclI allele in a homozygous variant had a higher prevalence of central adiposity, impaired glucose tolerance, diabetes mellitus and dyslipidemia [149]. Studies have also shown greater susceptibility to cardiovascular disease in men with the BclI haplotype [150,151].

Another well studied polymorphism of the GR gene is the N363S. N363S results from an A to G nucleotide change in codon 363, resulting to the substitution of asparagine (N) by serine (S). N363S has been associated with increased sensitivity to GCs in vivo [152], obesity, higher weight, and BMI in T2D patients [153]. Studies have also shown that High Density Lipoprotein (HDL) is positively associated with N363S polymorphism of the *NR3C1* gene, especially in Turkish female T2D patients [153]. In addition, Roussel et al. revealed that the N363S variant is associated with susceptibility to overweight in subjects with T2D [154]. The N363S polymorphism has been associated with reduced disposition index only in women and obesity [140,155]. Jewell et al. showed that the N363S SNP affected global gene regulation and altered both insulin signaling and inflammatory gene expression, suggesting an explanation for the correlation among metabolic syndrome, T2D, cardiovascular disease and N363S polymorphism [156]. In particular, N363S SNP carriers exhibit elevated levels of *HSD11B1*, and decreased levels of *Insulin-like Growth Factor 1 (IGF-1)* and *Insulin Receptor Substrate 1 (IRS1)* compared to non-carriers [156]. Furthermore, *CD163*, a predictor of increased risk of developing T2D [157] and *Toll-like Receptor 2 (TLR2),* a receptor involved in insulin resistance, inflammation, and diabetes [158], were found to be also elevated in N363S carriers in the presence of Dex [156].

Finally, GR-9β or A3669G (rs6198) is an A to G nucleotide substitution located in the 3′-Untranslated Region (UTR) of exon 9β. Studies have shown that 9β polymorphism results in an increased expression and stabilization of GRβ mRNA and leads to a relative GC resistance [159]. A3669G has been related to a lower risk of obesity in Europid women and a more favorable lipid profile in Europid men [160]. In a study on Italian patients with Cushing’s syndrome, 9β polymorphism has been shown to have a protective role, decreasing the risk of developing T2D [161]. Taken together, a great number of GR polymorphisms have been associated with metabolic abnormalities and increased risk of T2D, supporting further the important role of GR in T2D development. The association of GR polymorphisms with GC resistance/sensitivity as well as with T2D and metabolic indices are summarized in Table 2.

## 5. The Effects of Micro-RNAs on Glucocorticoid Receptor in Type 2 Diabetes Mellitus

MicroRNAs (miRNAs) consists of a family of non-coding RNAs with important functions in regulation of gene expression [162]. Dysregulation of miRNAs has been associated with a variety of diseases, including cardiovascular disease, kidney disease, and cancer [163]. Wang et al. investigated the expression levels of two stress-related circulating mi-RNAs, miR-18a and miR-34c, in PBMCs of T2D patients [164]. MiR-18a and miR-34c modulate central cell responsiveness to stress by targeting *GR* and *Corticotropin-Releasing Factor Receptor type 1 (CRFR1)* mRNA, respectively. Their study revealed that miR-18a and miR-34c expression levels were significantly different between T2D patients and healthy controls, suggesting that these miRNAs may play a role in vulnerability to insulin resistance and T2D [164]. In addition, Wang et al. have also shown that miR-192-3p is involved in fat accumulation and insulin sensitivity in the liver of diabetic mice by targeting GR, suggesting this miRNA as a potential therapeutic target for diabetes mellitus and fatty liver disease [165]. Taken together, the above data point out the importance of miRNAs, which are involved in GR signaling, in metabolic abnormalities and support further the role of GR in T2D development.

## 6. Glucocorticoid Receptor and Stevia Glycosides

*Stevia rebaudiana* Bertoni is a herb known for the high content of natural sweeteners in its leaves, namely stevioside and rebaudioside A, while steviol constitutes their major metabolite. Steviol glycosides are widely used today as non-caloric sweeteners because of their low Glycemic Index (GI) in patients with T2D, obesity, and metabolic syndrome [166]. Moreover, it has been suggested that steviol glycosides may also exert therapeutic benefit since they demonstrate anti-inflammatory, anti-hyperglycemic, anti-hypertensive, anti-tumor, anti-diarrheal, diuretic, and immunomodulatory activity [167,168,169,170]. Initial studies from Chang et al. claimed that stevioside presents GRE-mediated effect in mouse blood cells, implying that stevioside may be a GR modulator which could exert adverse effects on HPA axis and metabolism [171].

Panagiotou et al. investigated the possible GR modulatory activity of steviol glycosides in PBMCs and in human leukemic T-cells (Jurkat cells) [172]. Their results revealed that none of the tested compounds altered the expression of primary GR-target genes *(GILZ, FKPB5*), GR protein levels or GR subcellular localization in PBMCs. On the other hand, those compounds increased *GILZ* and *FKPB5* mRNA levels as well as GRE-mediated luciferase activity, inducing in parallel GR nuclear translocation in Jurkat cells [172]. Importantly, oral consumption of a product highly concentrated in steviol glycosides did not affect the *GILZ* and *FKBP5* expression and did not change the serum cortisol and ACTH levels. This study provides strong evidence that steviol and steviol glycosides exert GR-mediated effects in cancer Jurkat cells but do not exert any GR-dependent effects on normal human PBMCs and HPA axis [172].

Dusek et al. [173] investigated the interactions of steviol and stevioside with nuclear receptors, including GR, as well as their effect on expression of major cytochrome P450 genes (*CYPs*) which are drug-metabolizing enzymes, and they are regulated by GCs [174]. Their study indicated that stevia and stevioside cannot interact with GR in primary human hepatocytes but steviol moderately activate the pregnane X (PXR) and aryl hydrocarbon (AHR) receptors, resulting in the induction of their target genes *CYP3A4* and *CYP1A2,* indicating the potential of steviol to induce food-drug interactions in a GR-independent action [173]. Furthermore, Corcuff et al. investigated the metabolic effects of rebaudioside on HSD1 and HSD2 activities in nocturnal urine of healthy subjects who had ingested a commercially available sweetener containing rebaudioside A [175]. Their study revealed absence of significant effect of rebaudioside on HSD1 or HSD2 activities, supporting that rebaudioside should not promote metabolic syndrome via abdominal fat and local cortisol regeneration [175].

## 7. Concluding Remarks

Stress and depression increase the risk of T2D. T2D patients are characterized by an impaired negative feedback of the HPA axis (GC resistance) resulting in HPA axis hyperactivity and hypercortisolism. HPA axis hyperactivity is considered the biological link between stress, depression, and T2D-associated metabolic abnormalities. The continual HPA axis activation and the excess cortisol release exert detrimental effects on metabolism resulting in hyperglycemia. These include activation of glucose production and release, inhibition of glucose uptake, oxidation, and glycogen synthesis as well as impairment of insulin signaling in peripheral metabolic tissues such as liver, skeletal muscle, adipose tissue, and pancreas. Elucidation of the underlying molecular mechanisms revealed that GR is the key molecule which mediates the enhanced GC effects on metabolism (increased GC sensitivity). In particular, it has been shown that genes involved in various pathways in glucose metabolism are GR-primary target genes which are transcriptionally regulated by GR. In addition, GR polymorphisms have been associated with metabolic disturbances in T2D while dysregulation of micro-RNAs-known to target GR mRNA-has also been described, pointing out the role of GR in diabetes-associated metabolic abnormalities.

In contrast to increased GC sensitivity in metabolic tissues, there is impaired GR signaling in PBMCs in T2D which is associated with hyperglycemia, hyperlipidemia, and increased inflammation. Given that GR is similarly regulated in brain and immune cells, these data implicate that a reduced brain GR function may be a biological link among stress, HPA hyperactivity, hypercortisolism, and hyperglycemia in T2D. Hyperglycemia may promote a GR-resistant state in HPA axis negative feedback resulting in hypercortisolism, possibly via increased ROS and oxidative stress (factors known to impair GR function), creating a vicious cycle in T2D patients. Collectively, current evidence supports that GR plays a crucial role in T2D development, exerting cell type and tissue specific effects which result in either increased GC sensitivity or GC resistance. Modulation of GR signaling pathways by use of selective ligands, dissociating metabolic and anti-inflammatory actions of GR, may be a new therapeutic target for T2D.

## Figures and Tables

**Table 1:** Alterations of GR signaling parameters and evaluation of GC response in immune cells of T2D patients.

Patients/Sample	GR ^1^ Signaling Parameters	GC ^2^ Sensitivity Assay	GC Response	Ref.
T2D ^3^ patients vs. controls (PBMCs ^4^)	pGR-S211 protein levels ↓			[126]
	*GRβ* mRNA levels ↑			
	Basal *GILZ* mRNA levels ↓ Dex ^5^-induced *GILZ* mRNA levels ↓	Basal and Dex induced expression of GR-stimulated genes	Reduced GC sensitivity (GC resistance)	
	Basal *FKBP5* mRNA levels ↓ Dex-induced *FKBP5* mRNA levels ↓	Basal and Dex induced expression of GR-stimulated genes	Reduced GC sensitivity (GC resistance)	
T2D patients vs controls (Whole Blood Cells)	Less capable of producing IL-6 after LPS ^6^ stimulation Lower Dex concentration needed to inhibit LPS-induced IL-6	Dex inhibition of LPS-induced IL-6 production	Increased GC sensitivity	[127]

^1^ GR; Glucocorticoid Receptor, ^2^ GC; Glucocorticoid, ^3^ T2D; Type 2 Diabetes, ^4^ PBMCs; Peripheral Blood Mononuclear Cells, ^5^ Dex; Dexamethasone, ^6^ LPS; Lipopolysaccharide, ↑ increased levels, ↓ decreased levels.

**Table 2:** Association of GR polymorphisms with GC resistance/sensitivity as well as with T2D and metabolic indices.

GR ^1^ Polymorphisms	GC ^2^ Response	Refs	Metabolic Indices	Population	Ref.
ER22/23EK	GC resistance	[139]	total and LDL **^3^** cholesterol levels, fasting insulin levels ↓	Healthy elderly subjects	[139]
			insulin secretion, disposition index ↓	Women with normal/impaired glucose tolerance	[140]
			muscle strength and beneficial body composition ↑	Healthy subjects	[141]
Tth111I			basal and bedtime salivary cortisol levels ↑	Middle-aged men	[142]
ER22/23EK and Tth111I	GC resistance	[143]	fasting insulin levels, total and LDL cholesterol levels ↓	Elderly subjects	[143]
BclI	Increased GC sensitivity	[148]	body weight, BMI **^4^** ↑ abdominal obesity fasting plasma glucose, insulin, HOMA **^5^** ↑	Swedish men born in 1944	[144]
			salivary cortisol values after stimulation by a standardized lunch ↑ abdominal obesity	Middle-aged men	[145]
			fasting insulin, HOMA↑	Women with obesity	[146]
			total body fatness, insulin resistance ↑	Participants from CODAM ^8^ and the Hoorn study	[147]
			central adiposity, impaired glucose tolerance, diabetes mellitus, dyslipidemia	Patients with Addison’s disease	[149]
N363S	Increased GC sensitivity	[152]	obesity, weight, BMI, HDL **^6^** ↑	Patients with T2D	[153]
			overweight	Patients with T2D	[154]
			obesity	Non-diabetic white subjects of British descent	[155]
			mRNA levels *HSD11B1* ↑ mRNA levels of *IGF-1* ↓ mRNA levels of *IRS1* ↓	Healthy subjects	[156]
			disposition index ↓	Women with normal/impaired glucose tolerance	[140]
GR-9β	GC resistance	[159]	risk of obesity ↓ total cholesterol levels ↓ HDL↑	Europid women	[160]
			risk of developing T2D **^7^** ↓	Patients with Cushing’s syndrome	[161]

^1^ GR; Glucocorticoid Receptor, ^2^ GC; Glucocorticoid, ^3^ LDL; Low-Density Lipoprotein, ^4^ BMI; Body Mass Index, ^5^ HOMA; Homeostatic Model Assessment of Insulin Resistance, ^6^ HDL; High-Density Lipoprotein, ^7^ T2D; Type 2 Diabetes, ^8^ CODAM: Cohort on Diabetes and Atherosclerosis Maastricht, ↑ increased, ↓ decreased.

## Data Availability

Not applicable.
